# Supplementary material for: Patient-reported outcomes of joint-preserving surgery for moderate hallux rigidus: a 1-year follow-up of 296 patients from Swefoot
Source: Acta Orthop. 2020 Sep 25;92(1):109–13. doi: 10.1080/17453674.2020.1824762 (PMC7919882; doi:10.1080/17453674.2020.1824762)
Supplement: Supplemental Material [file IORT_A_1824762_SM0135.pdf]

OPEN ACCESS

Patient-reported outcomes of joint-preserving surgery for moderate hallux rigidus: a 1-year follow-up of 296 patients from Swefoot

Marcus E CÖSTER [1,2], Fredrik MONTGOMERY [1], and Maria C CÖSTER [1,3]

[1] Department of Clinical Sciences, Lund University; [2] Department of Orthopedics, Central Hospital in Växjö; [3] Department of Orthopedics, Skåne University Hospital in Malmö, Sweden
Correspondence: maria.coster@skane.se
Submitted 2020-04-07. Accepted 2020-09-01.

Background and purpose — Hallux rigidus (HR) may cause decreased range of motion, joint pain, and gait disturbances. There is a lack of evidence regarding the outcome of different surgical procedures for moderate HR. We report patient-reported outcomes after joint-preserving surgical procedures for moderate HR.

Patients and methods — We included 296 patients registered in Swefoot (Swedish national registry of foot and ankle surgery) who underwent primary surgery for moderate HR 2014 through 2018. We extracted information on anthropometrics, grading of HR, chosen surgical procedure, and patient-reported data including the PROMs SEFAS (summary score 0–48) and EQ-5D-3L (index 0–1) preoperatively and 1 year postoperatively.

Results — 115 patients underwent metatarsal decompression (i.e., Youngswick) osteotomy (YOT) and 181 underwent cheilectomy. The mean improvement in SEFAS score 1 year after surgery was 12 points (95% CI 10–13) for YOT and 10 points (CI 9–12) for cheilectomy. Also, EQ-5D improved in both groups. Patients who underwent YOT were more satisfied with the procedure (84% vs. 70 % for cheilectomy, p = 0.02).

Interpretation — Surgically treated patients with moderate HR improved after both YOT and cheilectomy, according to patient-reported data from Swefoot. Patients who underwent a YOT were more satisfied with their procedure. One possible explanation may be that more patients in the YOT group had a concomitant hallux valgus; however, we have no information on this.

Hallux rigidus (HR), which affects approximately 2% of the population, can be graded based on the severity of the disease (Lucas et al. 2015, Lam et al. 2017, Galois et al. 2020). Beeson et al. (2008) conducted a literature review of the existing multiple classification systems where the system promoted by Coughlin and Shurnas (2003) was deemed to have the most reliable grading.

Surgical treatment options can be joint-preserving and joint-sacrificing procedures. Several studies have shown positive outcomes for different surgical procedures (Coughlin and Shurnas 2003, Stevens et al. 2017, Coutts and Kilmartin 2019, Sidon et al. 2019, Slullitel et al. 2019), but there is a lack of consensus on what procedure should be chosen for a certain grade of HR (Galois et al. 2020). For moderate HR, when joint-preserving surgery is preferred, techniques such as cheilectomy, phalangeal and metatarsal osteotomies could be used (Coughlin and Shurnas 2003, Slullitel et al. 2016, Sidon et al. 2019, Galois et al. 2020). The evidence regarding which of these techniques should be used is, however, sparse. In Sweden most joint-preserving procedures are Youngswick osteotomies (YOT) and cheilectomies. YOT is a chevron-shaped decompression osteotomy of the first metatarsal where a slice of bone is removed in the dorsal arm of the osteotomy to achieve plantar and proximal displacement of the head. Most surgeons use a modified osteotomy, where the slice of bone being removed is smaller than what was originally described (Youngswick 1982, Gerbert et al. 2001). This procedure gives the possibility to address the hallux valgus deformity additionally. Even though studies have shown positive outcomes for both YOT and cheilectomy (Coughlin and Shurnas 2003, Sidon et al. 2019, Slullitel et al. 2019) there are few studies

© 2020 The Author(s). Published by Informa UK Limited, trading as Taylor & Francis Group, on behalf of the Nordic Orthopedic Federation. This is an Open Access article distributed under the terms of the Creative Commons Attribution License (http://creativecommons.org/licenses/by/4.0/), which permits unrestricted use, distribution, and reproduction in any medium, provided the original work is properly cited.

DOI 10.1080/17453674.2020.1824762

comparing these procedures. There is some evidence suggesting that cheilectomy has a higher frequency of revision surgery (Cullen et al. 2017), but no consensus on recommendations exists. Therefore, we analyzed patient-reported outcome for YOT and cheilectomy reported to Swefoot in patients with moderate HR. We hypothesized that YOT would have better outcomes than cheilectomy.

Patients and methods

Swefoot

We used data from Swefoot, a Swedish national quality registry of foot and ankle surgery. Swefoot was launched in 2014 and covers approximately 50% of all units performing foot and ankle surgery in Sweden. The registry contains baseline preoperative data; for example, sex, age, anthropometrics, comorbidities, and smoking habits. Patients are asked to complete 2 patient-reported outcome measures (PROMs): SEFAS (Self-reported Foot and Ankle Score) and EQ-5D 3L (EuroQol 5-dimensional 3 level version) before surgery, and 1 and 2 years after surgery. In addition to these PROMs patients are at the same time asked 4 questions regarding appearance, shoes, strength, and forefoot pain. After 1 and 2 years, they also complete 4 specific questions regarding their satisfaction with the result of the procedure and existence of any adverse events (Appendix, see Supplementary data). At the time of surgery, the surgeon reports the diagnosis, radiographic findings, severity of disease, type of surgical procedures, type of fixation, and postoperative routine.

Patient-reported outcome measures (PROMs)

SEFAS is a self-reported PROM specific for disorders in the foot and ankle, which has been described and validated for both the forefoot and hindfoot in previous publications (Cöster et al. 2012, 2014, 2018). A summary score is calculated based on the answers ranging from 0 (severe disability) to 48 (normal function). SEFAS covers different constructs, which are not reported separately in subscales. The most important of these constructs are pain, functional limitations, and quality of life (QoL).

EQ-5D is a generic PROM evaluating health-related quality of life (QoL) (EuroQol 1990) that is also validated and used in patients with foot and ankle disorders. We used the UK tariff to calculate an index score ranging from 0 to 1.0 where 1.0 represents full health.

Patients

We extracted data from Swefoot for all 623 reported patients who underwent primary surgery for moderate HR with YOT (including dorsal osteophyte resection) or cheilectomy between January 1, 2014 and December 31, 2018. To determine the severity of HR we used the grading system suggested by Coughlin and Shurnas where grade 0 represents mild disease and grade 4 advanced disease. We regarded grade 2 and 3 as moderate HR and included only patients in these grades. We excluded 327 patients who had not completed the PROMs both preoperatively and 1 year postoperatively (154 pre- and 173 postoperatively). These inclusion criteria resulted in 296 patients. Due to the large proportion of excluded patients we undertook a dropout analysis, which showed that preoperative age, sex, SEFAS score, and EQ-5D index were similar in both the study group and dropouts. Patients in the dropout group had slightly higher BMI, but in a sub-analysis this difference was equal for the YOT and the cheilectomy group.

Statistics

Data are reported as numbers and proportions (%), mean (SD), or median (range). We considered a probability of less than 5% as statistically significant and used 95% confidence intervals (CI) to describe uncertainty. Outcome is reported in the registry as the summary score for SEFAS and index for EQ-5D before and after surgery. Delta score and index is calculated as postoperative value minus preoperative value. The delta score, i.e., the absolute difference, could be without clinical relevance. Due to this we related the absolute difference to the minimally important change (MIC) for the PROMs. MIC reflects the smallest measured change in score that patients perceive as being important and defines a threshold when a treatment should be regarded as clinically relevant. The MIC value for the SEFAS in patients with forefoot disorders is 5 score points (Cöster et al. 2017). The MIC value for the EQ-5D in patients with foot and ankle disorders it is not defined, but in patients with back pain, another musculoskeletal disorder, the value is 0.173 (Johnsen et al. 2013). Group comparisons were performed using an independent-samples t-test for parametric data and Mann–Whitney U-test or chi-square test for non-parametric data. We used IBM SPSS Statistics version 24 (IBM Corp, Armonk, NY, USA) to perform the statistical analyses.

Ethics, data sharing, funding, and potential conflicts of interest

The study protocol was approved by the Ethical Review Board (Etikprövningsmyndigheten) in Sweden (reference number 2019-02733). The study was conducted in accordance with the Helsinki Protocol. The registration of data and the study were performed confidentially after patient consent and according to Swedish and EU data protection rules. Data may be accessible upon application to the registry.

The study was supported by grants from Herman Järnhardt foundations. The Swedish Foot and Ankle Registry (Swefoot) is financed by the Swedish Association of Local Authorities and Regions. The funders had no influence on the design of the study, the collection, analysis, and interpretation of data, on writing the manuscript, or on any other part of the study. The authors declare no conflict of interest.

Table 1. Preoperative baseline data presented as numbers with percentages unless otherwise specified

	Osteotomy (n = 115)	Cheilectomy (n = 181)	p-value
Mean age (SD)	55 (10)	58 (11)	
median (range)	60 (21–78)	55 (29–82)	0.04
Female sex	85 (74)	112 (62)	0.03
Mean BMI (SD)	26 (4)	27 (4)	0.2
Diabetes mellitus	5 (4)	8 (4)	1.0
Rheumatoid arthritis	5 (4)	6 (3)	0.3
Smoker	4 (4)	2 (1)	0.2
Quit smoking before surgery	7 (6)	12 (7)	
HR grade			0.7
2	47 (41)	70 (39)	
3	68 (59)	111 (61)	

Table 3. Patient-reported satisfaction. Values are numbers with percentages

	Osteotomy (n = 115)	Cheilectomy (n = 181)	p-value
**Satisfaction with outcome of surgery**			
Satisfied	97 (84)	127 (70)	0.006
Neither satisfied nor dissatisfied	6 (5)	16 (9)	
Dissatisfied	12 (10)	38 (21)	0.02
**Satisfaction with appearance of the feet**			
Preoperatively			
Satisfied	46 (40)	103 (57)	0.005
Dissatisfied	30 (26)	37 (20)	0.3
Postoperatively			
Satisfied	98 (85)	139 (77)	0.08
Dissatisfied	3 (3)	15 (8)	0.04

Table 2. Preoperative, postoperative and mean increase in SEFAS score and EQ-5D index from before surgery until the 1-year follow-up for respective procedure. Values are mean (SD) (95% CI)

	Osteotomy (n = 115)	Cheilectomy (n = 181)	Group comparison
SEFAS summary score			
preoperative	26 (7)	26 (8)	a
postoperative	38 (8)	36 (9)	1.5 (–0.6 to 3.5)
delta	12 (8) (10–13)	10 (9) (9.1–12)	1.2 (–0.7 to 3.2)
EQ-5D index			
preoperative	0.59 (0.3)	0.61 (0.3)	b
postoperative	0.80 (0.2)	0.77 (0.2)	0.04 (–0.01 to 0.09)
delta	0.21 (0.3) (0.15–0.26)	0.16 (0.3) (0.11–0.20)	0.05 (–0.02 to 0.12)

Delta is calculated as postoperative value minus preoperative value.
[a] p = 0.8
[b] p = 0.7

Results

Surgery was performed on 296 patients, 115 with a YOT and 181 with a cheilectomy. Both methods were used equally in most of the regions in Sweden except in the central part of Sweden where cheilectomy was the preferred method. In the YOT group the patients were younger and included more women compared with the cheilectomy group (Table 1). SEFAS summary score and EQ-5D index increased and the increase was statistically significant but also clinically relevant (based on MIC values) for both the YOT and cheilectomy group 1 year postoperatively compared with before surgery (Table 2). The CI of the changes by surgery all exceeded MIC, inferring that with more than 95% probability there is improvement exceeding MIC in the general population also. Postoperative scores and indices were similar in both groups (Table 2).

84% of patients in the YOT group reported that they were satisfied with their procedure compared with 70% in the cheilectomy group (p = 0.01). Also, a lower percentage of patients in the YOT group compared with the cheilectomy group reported dissatisfaction with the results (Table 3). Plantar forefoot problems were reported postoperatively among 3% in the osteotomy group and 8% in the cheilectomy group (p = 0.1).

Before surgery a higher percentage of patients in the YOT group compared with the cheilectomy group reported that they were dissatisfied with the appearance of their feet. Conversely, postoperatively more patients in the YOT group reported satisfaction with the appearance of their feet compared with the cheilectomy group (Table 3).

77% of patients in the YOT group were satisfied with the shoes they were able to wear postoperatively compared with 76% in the cheilectomy group. Also, 82% from the YOT group were satisfied with their postoperative foot strength compared with 79% in the cheilectomy group (p = 0.5).

Discussion

In this registry study we found that both YOT and cheilectomy resulted in improved foot-related pain and function and health-related QoL for patients with moderate HR. Both the SEFAS score and EQ-5D index increased more than their identified thresholds for clinical relevance. These results are in line with previous studies that have shown positive outcomes both short term and long term and suggest that both techniques are adequate to treat moderate HR surgically with positive effects evolving within 1 year after surgery (Waizy et al. 2010, Cullen et al. 2017).

When we started this study, we expected that the YOT and the cheilectomy group were identical and comparable in base-

line features such as age, percentage of women, anthropometrics, and presence of other foot and ankle disorders. We hypothesized that the YOT group should be more satisfied than the cheilectomy group after surgery. However, we found that patients in the YOT group were younger and included more women. When comparing the 2 surgical procedures we found similar improvement in PROMs in the 2 groups. However, we also found that a higher percentage of patients in the YOT group reported that they were satisfied with their surgery and that patients in the cheilectomy group were dissatisfied at a higher percentage. A possible explanation for this is that there are other aspects than PROMs can capture which also affect satisfaction with surgery. Another possible explanation may be that the YOT group contains proportionally more patients with concomitant hallux valgus (HV). The YOT can be used to decompress the joint with osteoarthritis and in the same procedure additionally to correct a HV deformity. There was a higher percentage of women in the osteotomy group and thus perhaps also more patients with concomitant HV (HV is more common in women than men; Coughlin and Jones 2007). Also, the fact that the patients in the YOT group were less satisfied with the appearance of their feet preoperatively could indicate that they also had HV and thus they would be more satisfied with surgery if this deformity was corrected as well. This would then also explain why these patients were satisfied at a higher grade with their appearance of their feet postoperatively. In summary, all these facts indicate that HV could be overrepresented in the YOT group. Thus, the 2 groups are not completely comparable. Although HR is a disease with strict diagnostic criteria the group of patients with HR is to some extent a mixed group between HR and HV. This must be considered when analyzing the results as it makes it possible that with the YOT we treat 2 conditions in these patients and thereby receive better results.

Swefoot does not specify HR patients with a concomitant HV. With the knowledge from this study we will adjust the registry to recognize these patients. We will also add a possibility for the surgeon to report additional corrective surgery.

Our hypothesis was that YOT would have better results than cheilectomy in moderate HR because YOT with dorsal osteophyte resection not only has immediate effect on ROM and thereby pain and gait function (as does cheilectomy), but also changes the length and angle of the metatarsal, which should also improve symptoms. In theory, these changes could reduce the risk for secondary surgery, but our 1-year follow-up time might be too short to capture such differences. Previous studies have used a 5-year follow-up regarding reoperation (Cullen et al. 2017, Slulittel et al. 2019). Future studies should include a longer follow-up time and have reoperation as an outcome to be able to address this question.

With decompression YOT there have been concerns regarding postoperative development of metatarsalgia due to the shortening of the metatarsal bone. We asked patients at the 1-year follow-up what degree of plantar forefoot problems they experienced and in fact tendencies were that the cheilectomy group experienced more symptoms. At the very least there was similar symptomatology in both groups. Although plantar forefoot problems are not directly transferrable to a diagnosis of metatarsalgia this is an indication that there were no obvious differences in experienced problems and thus the concerns regarding postoperative metatarsalgia after decompression osteotomy seems to be a minor issue when deciding on the procedure for moderate HR.

We did not present adverse events, revision rate, or secondary surgery because the registry is too new.

There are some limitations with this study. As this is a multicenter register study data reporting could vary between centers and surgeons. This could affect for example grading of HR as it is based on findings reported from the surgeon. Some centers might prefer one of the surgical procedures and combined with different experience of foot and ankle surgery this could affect outcomes. Furthermore, we are not able to make long-term statements due to the short follow-up time of 1 year. In Sweden, phalangeal osteotomies are not performed although this is a common procedure in several other countries. Hence, we are not able to include these procedures in the analysis.

The strengths of this study are the multicenter register design, the large sample size, and that we prospectively examine data collected in routine clinical practice. Another strength is that we have used a region-specific PROM, SEFAS, that is thoroughly evaluated in patients with foot and ankle disorders.

In conclusion, surgically treated patients with moderate HR improved within 1 year after surgery after both YOT and cheilectomy according to patient-reported outcomes, and the improvement was clinically relevant. In HR patients with a concomitant HV the YOT might be the better surgical method. This ought to be evaluated further in future studies.

Supplementary data

The Appendix is available as supplementary data in the online version of this article, http://dx.doi.org/10.1080/17453674.2020.1824762

MEC performed the statistical analyses and wrote the manuscript. FM assisted with the writing and statistics, as well as the planning of the study. MCC planned the study and supervised the writing.

Acta thanks Jan Willem Louwerens for help with peer review of this study.

Beeson P, Phillips C, Corr S, Ribbans W. Classification systems for hallux rigidus: a review of the literature. Foot Ankle Int 2008; 29(4): 407-14.

Cöster M, Karlsson M K, Nilsson J A, Carlsson A. Validity, reliability, and responsiveness of a self-reported foot and ankle score (SEFAS). Acta Orthop 2012; 83(2): 197-203.

Cöster M C, Bremander A, Rosengren B E, Magnusson H, Carlsson A, Karlsson M K. Validity, reliability, and responsiveness of the Self-reported Foot and Ankle Score (SEFAS) in forefoot, hindfoot, and ankle disorders. Acta Orthop 2014; 85(2): 187-94.

Cöster M C, Nilsdotter A, Brudin L, Bremander A. Minimally important change, measurement error, and responsiveness for the Self-Reported Foot and Ankle Score. Acta Orthop 2017; 88(3): 300-4.

Cöster M C, Rosengren B E, Karlsson M K, Carlsson A. Age- and gender-specific normative values for the Self-Reported Foot and Ankle Score (SEFAS). Foot Ankle Int 2018; 39(11): 1328-34.

Coughlin M J, Jones CP. Hallux valgus: demographics, etiology, and radiographic assessment. Foot Ankle Int 2007; 28(7): 759-77.

Coughlin M J, Shurnas P S. Hallux rigidus: grading and long-term results of operative treatment. J Bone Joint Surg Am 2003; 85-A(11): 2072-88.

Coutts A, Kilmartin T E. Dorsiflexory phalangeal osteotomy for grade ii hallux rigidus: patient-focused outcomes at eleven-year follow-up. J Foot Ankle Surg 2019; 58(1): 17-22.

Cullen B, Stern A L, Weinraub G. Rate of revision after cheilectomy versus decompression osteotomy in early-stage hallux rigidus. J Foot Ankle Surg 2017; 56(3): 586-8.

EuroQol Group. EuroQol–a new facility for the measurement of health-related quality of life. Health Policy 1990; 16(3): 199-208.

Galois L, Hemmer J, Ray V, Sirveaux F. Surgical options for hallux rigidus: state of the art and review of the literature. Eur J Orthop Surg Traumatol 2020; 30(1): 57-65.

Gerbert J, Moadab A, Rupley K F. Youngswick–Austin procedure: the effect of plantar arm orientation on metatarsal head displacement. J Foot Ankle Surg 2001; 40(1): 8-14.

Johnsen L G, Hellum C, Nygaard O P, Storheim K, Brox J I, Rossvol I, Leiseth G, Grotle M. Comparison of the SF6D, the EQ5D, and the Oswestry Disability Index in patients with chronic low back pain and degenerative disc disease. BMC Musculoskeletal Dis 2013; 14: 148.

Lam A, Chan J J, Surace M F, Vulcano E. Hallux rigidus: how do I approach it? World J Orthop 2017; 8(5): 364-71.

Lucas D E, Hunt K J. Hallux rigidus: relevant anatomy and pathophysiology. Foot Ankle Clin 2015; 20(3): 381-9.

Sidon E, Rogero R, Bell T, McDonald E, Shakked R J, Fuchs D, Daniel J N, Pedowitz D I, Raikin S M. Long-term follow-up of cheilectomy for treatment of hallux rigidus. Foot Ankle Int 2019; 40(10): 1114-21.

Slullitel G, Lopez V, Seletti M, Calvi J P, Bartolucci P, Pinton G. Joint preserving procedure for moderate hallux rigidus: does the metatarsal index really matter? J Foot Ankle Surg 2016; 55(6) 1143-7.

Slullitel G, Lopez V, Calvi J P, D’Ambrosi R, Usuelli F G. Youngswick osteotomy for treatment of moderate hallux rigidus: thirteen years without. arthrodesis. Foot Ankle Surg 2019; S1268-7731(19)30208-5.

Stevens J, de Bot R, Hermus J P S, van Rhijn L W, Witlox A M. Clinical outcome following total joint replacement and arthrodesis for hallux rigidus: a systematic review. JBJS Rev 2017; 5(11): e2.

Waizy H, Czardybon M A, Stukenborg-Colsman C, Wingenfeld C, Wellman M, Windhagen H, Frank D. Mid- and long-term results of the joint preserving therapy of hallux rigidus. Arch Orthop Trauma Surg 2010; 130: 165-70.

Youngswick F D. Modifications of the Austin bunionectomy for treatment of metatarsus elevatus associated with hallux limitus. J Foot Surg 1982; 21: 114-16.
